# Respiratory Rate Oxygenation (ROX) index as predictor of high flow nasal cannula in pediatric patients in pediatric intensive care unit

**DOI:** 10.1186/s12890-024-03029-2

**Published:** 2024-05-02

**Authors:** Irene Yuniar, Antonius Hocky Pudjiadi, Rismala Dewi, Yogi Prawira, Niken Wahyu Puspaningtyas, Tartila Tartila, Sharfina Fulki

**Affiliations:** Department of Child Health, Cipto Mangunkusumo Hospital, Jakarta, Indonesia

**Keywords:** HFNC, ROX index, Pediatrics

## Abstract

**Background:**

High-flow nasal cannula (HFNC) is often used in pediatric populations with respiratory distress. In adults, the respiratory-rate oxygenation (ROX) index is used as a predictor of HFNC therapy; however, children have age-associated differences in respiratory rate, thus may not be applicable to children. This study aims to find the reliability of ROX index and modified P-ROX index as predictors of HFNC therapy failure in pediatric patients.

**Methods:**

Subjects in this analytical cross-sectional study were taken from January 2023 until November 2023 in Cipto Mangunkusumo Hospital. Inclusion criteria are children aged 1 month to 18 years with respiratory distress and got HFNC therapy. Receiver operating characteristics (ROC) analysis was used to find mP-ROX index cutoff value as a predictor of HFNC failure. The area under curve (AUC) score of mP-ROX index was assessed at different time point.

**Results:**

A total of 102 patients, with 70% of the population with pneumonia, were included in this study. There are significant differences in the ROX index between the successful and failed HFNC group therapy (*p* < 0.05). This study suggests that mP-ROX index is not useful as predictor of HFNC therapy in pediatrics. While ROX index < 5.52 at 60 min and < 5.68 at 90 min after HFNC initiation have a sensitivity of 90% and specificity of 71%, sensitivity of 78% and specificity of 76%, respectively.

**Conclusion:**

mP-ROX index is not useful as a predictor of HFNC therapy in pediatrics. Meanwhile, ROX index at 60 min and 90 min after initiation of HFNC is useful as a predictor of HFNC failure.

## Introduction

High-flow nasal cannula (HFNC) has been widely used as a non-invasive respiratory support for patients with acute respiratory distress [1, 2]. While endotracheal intubation or other invasive mechanical ventilation is the mainstay intervention of respiratory distress, it is associated with higher morbidity and mortality, risk of nosocomial infections, lung and airway trauma, duration of admission, and sedation-related complications [1, 3]. HFNC gives advantages in terms of humidification, oxygenation, CO_2_clearance, and work of breathing than conventional oxygen therapy. Higher oxygen flow can lead to nasopharyngeal dead space washout, decreased nasal mucosa resistance, increased oxygenation, and prevent atelectasis [3]. Although the use of HFNC in the pediatric population remains poorly understood, based on observational and randomized clinical trials done in infants with bronchiolitis, there was a reduction in the number of patients who needed intubation [4–6].

The efficacy and safety of HFNC made its use not only in pediatric intensive care unit (PICU), but also in the emergency department, and general ward. Although proper and close monitoring by trained staff is still necessary. The patient’s response to HFNC therapy was monitored in vital signs, work of breathing, and FiO_2_need [3]. A standard parameter to evaluate HFNC failure in adults is the SF ratio, which is the ratio of SpO_2_ to FiO_2_ [7]. Another parameter as a predictor of HFNC therapy that is widely used in adult patients is respiratory rate oxygenation (ROX) index, defined as the ratio of SF to the respiratory rate (RR) [8]. Roca et al. evaluated the ROX index in the adult population with pneumonia, cut-off value > 4.88 at 12 h after HFNC therapy, with a sensitivity of 70% and specificity of 72.4% [8]. Yildizdas et al. modified ROX index for the pediatric population (Pediatric-ROX index) by using respiratory rate z-score instead of RR in the formula. The study showed that P-ROX index can be used to predict the risk of HFNC failure in pediatric patients [5]. Studies on predictors of HFNC therapy in pediatric population still shows inconsistent results [9]. This study aims to find the reliability of the ROX index and modified P-ROX index as predictors of the need for intubation in HFNC therapy failure in pediatric patients.

## Methods

### Study design

This analytical cross-sectional study was conducted at a national referral hospital from January 2023 until November 2023. Patients aged one month to 18 years with respiratory distress of any etiology who were admitted to the pediatric ward, emergency department (ED), and pediatric intensive care unit (PICU) and who received HFNC therapy were included in this study. Exclusion criteria included intubated patients for diagnostic or therapeutic aims, unstable hemodynamics, patients with decreased level of consciousness or apnea patients, patients with neuromuscular disease, and patients with congenital facial abnormalities. Data was taken from medical records, patient’s characteristics (sex, weight), HFNC indication, SF ratio (SpO_2_/FiO_2_), and vital signs. ROX index was analyzed using SF/RR. In this study, mP-ROX was calculated using the respiratory rate percentile based on the Fleming chart [10]. The modified P-ROX index was defined as the ratio of SF to respiratory rate percentile based on Fleming chart. The estimation of sample size was based on the expected sensitivity (85%) and prevalence of failed HFNC therapy in respiratory distress children, which was 24.6%. The final sample size was 97, including 10% dropout, to provide sufficient power for the study.

### Study ethics

The study was approved by The Ethics Committee of the Faculty of Medicine, University of Indonesia—Cipto Mangunkusumo Hospital number: KET-

148/UN2.F1/ETIK/PPM.00.02/2022. Informed consent was given from the parents.

### Protocol

**Fig. 1:**
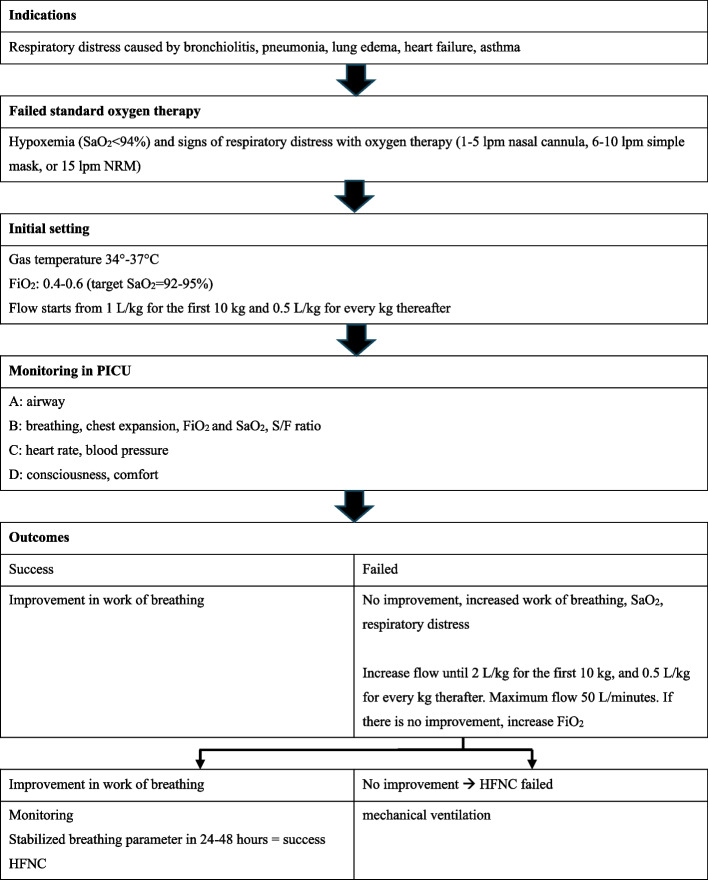
HFNC protocol

HFNC therapy using Airvo 2 Optiflow™ high flow therapy system (Fisher and Paykel). The attending physician was not informed of the mP-ROX index. Thus, failed cases were decided by the attending physician based on clinical judgment. The mP-ROX index and ROX index were assessed before initiation of HFNC treatment, then at 60 min, 90 min, and 24 h after HFNC treatment. Figure 1 represents the HFNC protocol used in this study. Success HFNC is defined as patients with stabilized breathing patterns in 24–48 h after initiation of HFNC.

### Statistical analysis

Data analysis was done with IBM *Statistical Package for the Social Sciences* (SPSS) version 23. Data was analyzed using the Kolmogorov–Smirnov test for normality test. Normally distributed data was presented in mean and standard deviation (SD), and non-normal distribution was shown in median (interquartile range). Receiver operating characteristics (ROC) and area under curve (AUC) analysis were used to determine the accuracy of the HFNC predictor (ROX and mP-ROX index). Youden index, which is the sum of sensitivity – (1 – specificity), is used to determine the best cutoff point for each time point. The sensitivity and specificity of each index were analyzed with a 95% confidence interval (CI). *P* value < 0.05 was considered statistically significant.

## Results

This study involved a total sample of 102 pediatric patients undergoing HFNC treatment in Cipto Mangunkusumo Hospital, which was taken from medical records for 11 months. The general characteristics of the sample study are described in Table 1. There were 92 patients (90%) with successful HFNC treatment that did not need NIV/mechanical ventilation. There is no significant difference in sex, weight, length of stay in PICU, and length of stay in hospital in this study. Most of the patients who responded with HFNC treatment aged younger than patients who failed HFNC treatment, but there are no significant differences. Pneumonia was the most common HFNC indication in this study. The in-hospital mortality in the successful group was 14 patients (13.7%), due to comorbid conditions. Patients with no improvement with HFNC treatment had a longer length of stay in the hospital (24 (2–148) days vs. 32.5 (2–63) days).

**Table 1:** General characteristics of sample study

Characteristics	Success (*n* = 92)	Failed (*n* = 10)	*p*-value
**Sex, n (%)**			0.578
Boy	44 (43.1)	5 (4.9)	
Girl	48 (47.1)	5 (4.9)	
**Weight (kg), median (range)**	8.8 (3.1–65)	10 (3.7–52)	0.562
**Age (months), median (range)**	12 (1–204)	42 (1–192)	0.477
**HFNC indication, n (%)**			
Pneumonia	72 (70.6)	9 (8.8)	
Sepsis	4 (3.9)		
Asthma	1 (1)		
Bronchiolitis	2 (2)		
Post extubation	2 (2)		
Lung Oedema	4 (3.9)		
Others	7 (6.9)	1 (1)	
**Outcomes, n (%)**			0.004
Alive	78 (76.5)	4 (3.9)	
Mortality	14 (13.7)	6 (5.9)	
**Length of stay in PICU (days), median (range)**	4.5 (0–273)	3.5 (0–63)	0.443
**HFNC duration (days), median (range)**	4 (1–27)	1 (0–31)	0.001
**Length of stay in hospital (days), median (range)**	24 (2–148)	32.5 (2–63)	0.823

**Fig. 2:**
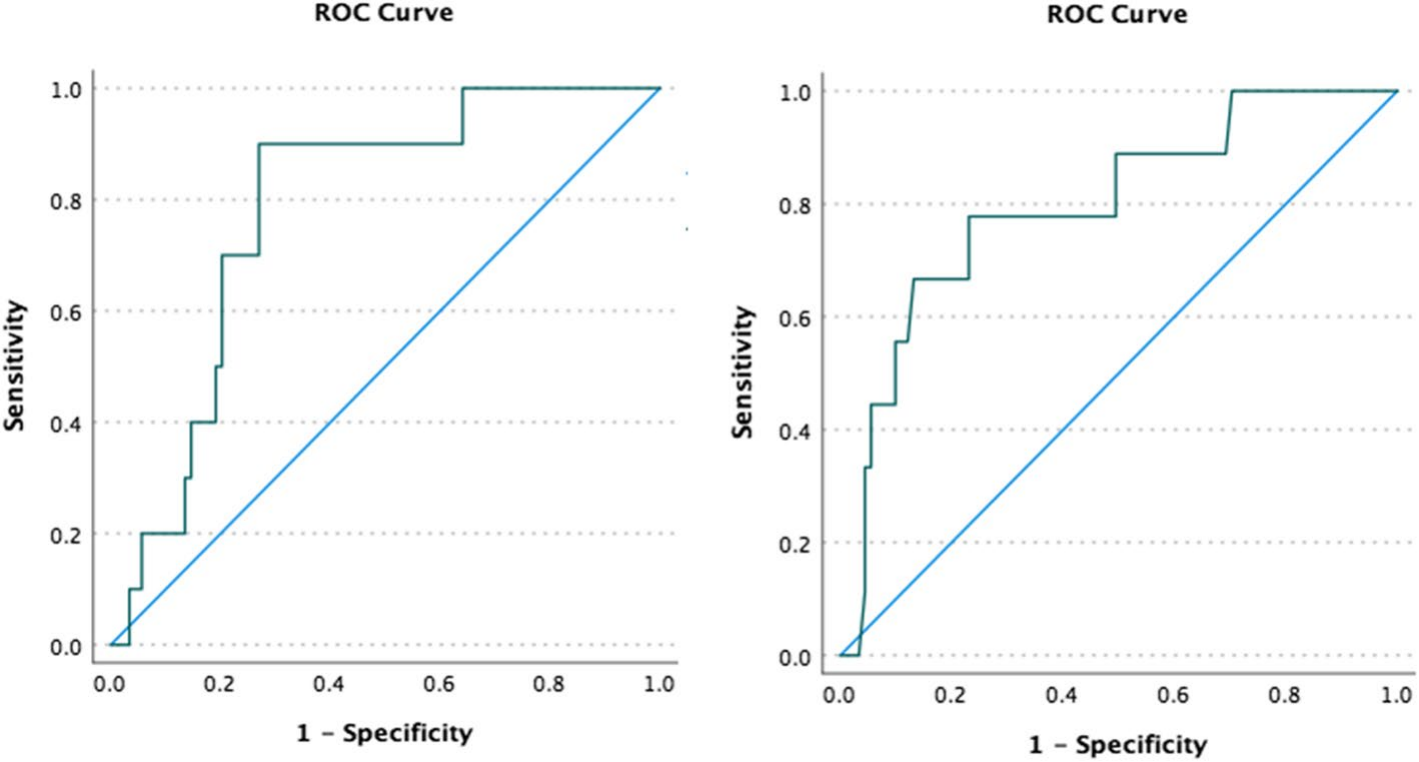
Receiver operating characteristic curve of ROX index. **a** 60 min after initiation of HFNC therapy. **b** 90 min after initiation of HFNC therapy. ROX, respiratory rate-oxygenation. HFNC, high-flow nasal cannula

This study observed ROX and mP-ROX index as predictors of HFNC treatment. S/F ratio, ROX, and mP-ROX index were assessed at different time points in pediatric patients with HFNC therapy: at initial therapy, 60 min, 90 min, and 24 h after initiation. HFNC assessment and monitoring on both the successful and failed groups are shown in Table 2. There was no significant difference in the initial score of both groups. However, there is a significant difference in ROX index score on 60 and 90 min after HFNC therapy. Blood gas analysis was done 60 min and 24 h after initiation; there was a higher PaO2 value in the successful group than in the failed group, but it is not statistically significant. This study has no failed cases of HFNC therapy after 24 h of use. The mean time to initiation of NIV/intubation was 12.2 h in failed cases, ranging from 7 to 22 h of HFNC use. Table 3 showed significant differences in ROX and mP-ROX index correlation in 60 and 90 min after initiation of HFNC. ROX index cutoff value on initial, 60 min, and 90 min after initiation of HFNC therapy was shown in Table 4. ROX index has good sensitivity and specificity scores on initial, 60 min, and 90 min of therapy (Fig. 2).

**Table 2:** HFNC assessment and monitoring in 60 min, 90 min, and 24 h after initiation

Characteristics	Success (*n* = 92)	Failed (*n* = 10)	*p*-value
Initial S/F ratio	235.30 ± 81.96	206.73 ± 61.86	0.257
Initial mP-ROX index	7.27 (2.34 – 44)	6.98 (2.34 – 10.31)	0.271
Initial ROX index	5.53 ± 2.17	4.53 ± 1.5	0.139
**Initial HFNC parameter**			
FiO_2_	40 (21 – 100)	47.5 (30 – 70)	0.524
Flow (L/minutes)	17 (6 – 50)	16 (8 – 40)	0.529
**mP-ROX index score**			
60 min	7.13 ± 2.95	6.29 ± 1.72	0.380
90 min	7.60 (0.47 – 18.33)	6.63 (2.08 – 15)	0.326
**ROX index score**			
60 min	6.58 ± 2.21	4.55 ± 1.28	0.006
90 min	7.42 ± 2.32	4.77 ± 2.16	0.001
**PaO**_**2**_** (60 min)**	104.59 ± 64.77	91.34 ± 52.14	0.348

*ROX* Respiratory rate-oxygenation, *mp-ROX* modified pediatric respiratory rate-oxygenation

**Table 3:** ROX and mP-ROX index correlation

mP-ROX and ROX index	r	*p*-value
Initial	0.090	0.349
60 min	0.536	0.000
90 min	0.481	0.000

*ROX* Respiratory rate-oxygenation, *mp-ROX* modified pediatric respiratory rate-oxygenation

**Table 4:** Diagnostic accuracy of ROX index

ROX index	**Sensitivity**	**Specificity**	**Cutoff value**	**AUC**
Initial	0.80	0.533	< 5.35	0.644
60 min	0.90	0.708	< 5.52	0.785
90 min	0.78	0.758	< 5.68	0.797

*ROX* Respiratory rate-oxygenation, *AUC* area under curve

## Discussion

This study examined the use of the mP-ROX index and ROX index as predictors of HFNC failure performed in patients aged 1 month to 18 years who received HFNC therapy with various etiologies. This study found that the ROX index is a parameter that can be used to assess HFNC failure in children, apart from clinical assessment. In Table 1, there are no differences in general characteristics between the two groups. However, there was a significant difference in the duration of HFNC use, whereas in the successful group, the duration of HFNC use was found to be longer. The duration of HFNC use was also significantly longer in the successful group in a study on bronchiolitis in infants [11–13]. This is due to switching to mechanical ventilation therapy in failed groups, which was associated with a longer length of stay [14, 15]. After 24 h of HFNC monitoring with a stabilized pattern of breathing, there were 14 patients (13.7%) who died in the successful group. Patients experienced deterioration after removal of HFNC treatment mostly due to comorbid conditions such as immunocompromised, malnutrition, and malignancy. In other cases, one patient had cyanotic congenital heart disease. However, there is a difference in initial S/F ratio between the alive and mortality group, although not significantly different. This could explain futhermore about the mortality in successful group.

There was no significant difference in blood gas analysis (BGA) results at 60 min between both groups. This shows that BGA examination does not always correlate with the patient's clinical condition. Routine sampling in the evaluation of HFNC therapy is also less effective and increases parental anxiety, child pain, and costs.

The severity of disease was assessed with S/F ratio, mP-ROX index, and ROX index was not found to be significantly different in the two groups (Table 2). The S/F ratio is a valuable and easy tool to assess the severity of respiratory distress. In the successful group, the S/F ratio was higher than the failed group, indicating that there is a slight difference in the hypoxemia severity. There was no difference in the initial HFNC settings, and there was no difference in the severity of respiratory distress. Lemiale et al. suggested that the ROX index can be used as a risk stratification for intubation to differentiate patients who would likely fail HFNC therapy [16].

Assessment with the mP-ROX index at 60 and 90 min did not show a significant difference between the two groups. However, there was a significant difference in the assessment with the ROX index in the success and failure groups (*p* < 0.05). It can be concluded that the ROX index is more reliable in assessing failure of HFNC therapy. The mP-ROX index is a novel parameter developed in this study. However, it showed poor diagnostic value in pediatric patients. Respiratory rate based on Fleming chart was used instead of normal respiratory rate in adults. As far as this research is concerned, there are only two studies regarding the P-ROX index as a predictor of HFNC therapy failure in the pediatric population. Yildizdas et al. used the respiratory rate z-score compared to the patient's respiratory rate and found a P-ROX index above 66.7 at the 24th hour as the cut-off value [5]. Saelim et al. found that the P-ROX index could be a helpful predictor in assessing failure of HFNC therapy using age-associated respiratory rate [12]. They found a P-ROX index < 132 at 12 h after HFNC with sensitivity of 63% and specificity of 88%. However, Kim et al. suggested that using the median RR rather than the respiratory z-score in the P-ROX index showed a better diagnostic value in pediatric patients with hypoxic respiratory failure [17].

Table 3 showed that there was no correlation between the initial mP-ROX and ROX index, but a moderate correlation was found at 60 and 90 min. Correlation test was done because both tests gave significantly different results. This indicates that the mP-ROX index has not proven useful in determining HFNC failure in children. In contrast to research by Yildizdas et al. which states that the P-ROX index can be used as a good predictor for failure of HFNC therapy in the pediatric population [5]. However, this study used the respiratory rate percentile based on the Fleming chart to calculate the mP-ROX index rather than the respiratory z-score.

**Fig. 3:**
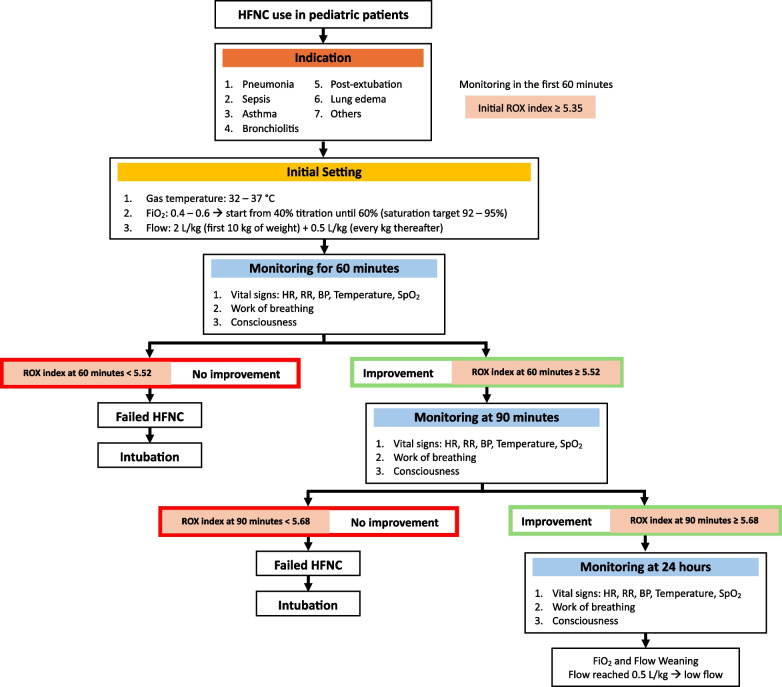
Pediatric HFNC management chart

Based on Table 4, we created an HFNC management chart for clinicians to determine the failure of HFNC therapy in children (Fig. 3). This study evaluated the ROX index at several time points: initial, 60th minute, 90th, and 24th hour. It was found that the 60th-minute ROX index with a cutoff value < 5.52 (AUC 0.785) had a sensitivity of 0.9 and a specificity of 0.708. The 90th-minute ROX index with a cut point < 5.68 (AUC 0.797) had a sensitivity of 0.78 and a specificity of 0.758. This shows that the ROX index is a good predictive factor for HFNC therapy in pediatric patients. In this study, after 24 h of initiation of HFNC use, no HFNC failure was found, with a duration of HFNC use of 4 (1 – 27) days. This is supported by several studies that suggest that failure generally occurs 5 to 12 h after initiation of HFNC therapy. This illustrates the importance of constant monitoring during HFNC therapy to prevent clinical deterioration [11, 18].

In this study, PICU length of stay was shorter in the failure group than in the success group. The shorter duration can be due to the severity of the disease, causing death cases. Meanwhile, the length of stay at the hospital was found to be longer in the failure group due to the use of mechanical ventilation. This was supported by Kannikeswaran et al. and Nascimento et al. who found that the duration of hospitalization was longer in the group that received positive pressure ventilation or in the group that failed HFNC [11, 13].

This study had few limitations, including a relatively small sample size and a wide range of age, thus may limit the interpretability of the data. However, this study had sufficient power to demonstrate the accuracy of the predictors to the general population. Further studies with larger population are nescessary to validation of the data.

## Conclusion

Based on this study, the mP-ROX index is not useful as a predictor of HFNC failure in pediatric patients. ROX index score on the 60th and 90th minute of HFNC therapy can be used significantly as a determinant of HFNC failure in addition to the patient's clinical assessment. Invasive blood gas analysis examinations in children are not recommended as clinical support because they are not statistically significant. This research is helpful in reducing invasive procedures, costs, and pain in children and reducing anxiety in parents regarding repeated blood draws. The use of the ROX index in HFNC monitoring with specific cutoff value at 60 and 90 min can be used as a predictor of HFNC failure.

## Data Availability

The datasets used and/or analyzed during the current study are available from the corresponding author upon reasonable request.

## Abbreviations

ROX: Respiratory-rate oxygenation
HFNC: High-flow nasal cannula
mP-ROX: Modified pediatric respiratory-rate oxygenation
AUC: Area under curve
ROC: Receiver operating characteristics
PICU: Pediatric Intensive Care Unit
ED: Emergency department
CO_2_: Carbon dioxide
FiO_2_: Fraction of inspired oxygen
SaO_2_: Arterial oxygen saturation
PaO_2_: Arterial oxygen pressure
SPSS: Statistical Package for the Social Sciences
SD: Standard deviation
CI: Confidence interval
